# Laser-Tracker-Based Robot Pose Measurement Using PSD Spot Sensing and Multi-Sensor Fusion with Simulation Validation

**DOI:** 10.3390/mi17030290

**Published:** 2026-02-26

**Authors:** Suli Wang, Jing Yang, Xiaodan Sang

**Affiliations:** College of Physics and Telecommunication Engineering, Zhoukou Normal University, No. 6, Middle Section of Wenchang Avenue, Chuanhui District, Zhoukou 466001, China

**Keywords:** laser tracker, robot pose measurement, interferometry, position sensitive detector, sensor fusion, tracking control

## Abstract

Accurate measurement of robotic pose is indispensable for large-scale precision manufacturing and robotic calibration, particularly because traditional robotic kinematic models often fall short owing to environmental disturbances and structural uncertainties. Laser tracker systems offer high-precision, large-volume measurement capabilities and are therefore appealing as external references for robot pose estimation; however, their practical efficacy is heavily reliant on optical tracking stability, sensor noise levels, and system robustness. This paper introduces a laser tracker-based framework for measuring robot pose, which integrates PSD-based optical spot sensing, multi-sensor fusion, and simulation-based system analysis. A prototype PSD sensing subsystem has been developed utilizing analog signal conditioning, high-speed A/D sampling, and FPGA-based centroid computation. Bench experiments validate the linearity, geometric sensitivity, and robustness of the PSD sensing chain under controlled spot translations and various ambient illumination conditions. Results demonstrate that the PSD response is nearly linear within a ±0.9 mm spot displacement and that the implementation of an interference optical filter significantly enhances measurement repeatability under background light. At the system level, a comprehensive simulation framework is established wherein PSD measurements are fused with inertial and encoder data via an extended Kalman filter. The simulations explore the effects of process noise tuning, time synchronization, systematic error sources, and control strategies on pose estimation accuracy. Ranging-related effects and error-compensation mechanisms are analyzed within the context of modeling and simulation, providing insights into the interferometric ranging principle underlying the complete laser tracker system. The validation of the prototype alongside simulation results demonstrates that PSD-based optical tracking, combined with multi-sensor fusion and layered error compensation, can effectively improve robustness and positional accuracy. The proposed framework offers valuable guidance for the development and phased validation of laser tracker-oriented robot pose measurement systems in complex industrial environments.

## 1. Introduction

Industrial robots are increasingly deployed in tasks where geometric accuracy is as critical as productivity: aircraft and ship assembly, large tooling alignment, automated drilling/riveting, precision welding, and additive manufacturing of large structures [1,2,3]. In these scenarios, the nominal kinematic model of a robot is rarely sufficient. Errors accumulate from manufacturing tolerances, joint backlash, thermal deformation, structural compliance, and time-varying disturbances such as vibration or air turbulence. In practical applications, the required geometric accuracy varies by industry. In aircraft assembly and large-scale tooling alignment, positioning accuracies of 0.1–0.3 mm over several meters are typically required [4,5,6]. Automated drilling and riveting processes often demand sub-millimeter accuracy (approximately 0.05–0.2 mm) to ensure hole alignment and fastener quality. In robotic welding and additive manufacturing of large structures, acceptable positioning errors are typically below 0.5 mm [7,8,9], whereas high-end precision manufacturing and calibration tasks may require accuracies approaching or exceeding 0.1 mm [10,11]. For high-precision applications, pose errors of a few millimeters or even sub-millimeters can translate into unacceptable process defects, such as hole misalignment in drilling and riveting, excessive weld seam deviation, poor mating of large structural components, increased residual stress, and degradation of dimensional consistency in additive manufacturing. Consequently, reliable robot pose measurement and calibration remain a central topic in robotics and metrology.

Laser trackers are widely used for large-scale three-dimensional measurement [12,13,14,15] because they can provide absolute distance and angular measurements over meter-level workspaces. In contrast to local sensors (e.g., cameras or proximity sensors), a tracker can cover a large volume with a single station and can support dynamic tracking of a cooperative target [16,17,18]. For robot pose metrology, this capability is attractive: the tracker can serve as an external reference to evaluate and correct robot motion, and it can also support in situ measurement when the workpiece or tooling cannot be moved to a coordinate measuring machine [19,20,21,22].

In addition to experimental validation, simulation studies play an important role in analyzing robot positioning accuracy [23,24,25,26]. Simulations enable systematic investigation of motion trajectories, sensor noise, delay effects, and error propagation under controlled, repeatable conditions that are often difficult to isolate in physical experiments [27,28,29,30]. Recent work has demonstrated that simulation-based analysis can yield reliable insights into robot motion behavior and positioning accuracy, providing valuable guidance for system design and parameter tuning prior to real-world deployment [31].

For example, in [20], the authors analyzed robot motion trajectories and positioning accuracy using simulation software, highlighting the effectiveness of simulation as a complementary tool to experimental measurement.

Despite their advantages, applying a tracker to robot pose estimation involves practical challenges that are sometimes obscured in high-level descriptions. First, accurate ranging is necessary but not sufficient: the optical path must remain stable and the tracker must maintain lock on the target. Second, the returned beam must be reliably sensed in the receiver plane with sufficient signal-to-noise ratio, even under varying ambient illumination. Third, the tracking actuator and control loop must be tuned to avoid oscillation, drift, or loss of lock during dynamic motion. Finally, a robot pose estimate often benefits from additional information sources: encoder feedback provides joint angle consistency, while inertial sensing improves short-term dynamic observability. A single-sensor solution may be fragile when line-of-sight is interrupted or when environmental disturbances dominate.

This work is organized around the idea that a robust pose measurement system should be developed and validated from the sensing chain upward. We first focus on prototype-level verification of the spot sensing and processing chain based on a pillow-type 2D PSD, analog conditioning, A/D sampling, and FPGA computation. We then build a simulation suite that integrates the prototype’s sensing concept with multi-sensor fusion, error modeling and compensation, controller comparison, and dynamic environment adaptation. Rather than treating simulations as independent, we use them to stress-test the kinds of errors and delays that the prototype would encounter in real deployment.

The main contributions of this paper can be summarized as follows: (1) a hardware and embedded implementation scheme for PSD-based spot sensing suitable for real-time tracking, including centroid computation on FPGA; (2) bench experiments that quantify PSD linearity over a controlled translation range, evaluate sensitivity to the laser head–PSD distance, and demonstrate repeatability improvement using an interference optical filter; (3) a simulation suite that fuses PSD, IMU, and encoder measurements using an EKF and evaluates convergence, noise robustness, and the importance of time synchronization; (4) systematic error modeling and compensation simulations covering temperature drift, coordinate transformation error, target sphere manufacturing deviation, and base vibration; and (5) control algorithm comparisons (PID, LQR, fuzzy) and dynamic environment adaptation demonstrations (obstacle avoidance, illumination compensation, humidity compensation).

## 2. Measurement Principle and Modeling

A laser tracker generally employs a spherical measurement model to reconstruct a three-dimensional point in a global coordinate frame. The measured quantities include a line-of-sight distance, denoted by *L*, and two angles, commonly referred to as the azimuth and the elevation. In accordance with the ideal spherical model, the Cartesian coordinates can be expressed as follows: In the given context, *x* is equivalent to *Lcos*(*θ*)*cos*(*φ*), *y* is equivalent to *Lcos*(*θ*)*sin*(*φ*), and z is equivalent to *Lsin*(*φ*), where θ and φ represent elevation and azimuth angles, respectively. In practice, the coordinate transformation also involves tracker base alignment, encoder scale factors, and offsets that must be calibrated.

Ranging can be implemented via interferometry. In an interferometric scheme, the displacement of the optical path is directly proportional to the number of interference fringes (or phase increments) observed when the path length undergoes a change. Interferometry is a superior method to purely incremental encoders in that it provides a direct optical displacement reference. This is of particular value for high-accuracy metrology. However, interferometric ranging is sensitive to environmental conditions (e.g., refractive index changes in air), requiring compensation based on temperature, pressure, and humidity when high accuracy is required.

For dynamic tracking, maintaining optical alignment is essential. In our prototype concept, a two-dimensional position sensitive detector (PSD) is used to sense the position of the returned beam spot on the receiver plane. A pillow-type PSD provides continuous analog output proportional to the spot location, enabling fast feedback for tracking mirror actuation. Let $U_{X}1,U_{X}2,U_{Y}1,$ and $U_{Y}2$ denote the digitized signals corresponding to the four electrodes. The spot centroid (CoG) coordinates can be computed using normalized difference-over-sum formulas. A representative form is: $X=\left(\left(U_{X}2+U_{Y}2\right)-\left(U_{X}1+U_{Y}1\right)\right)/\left(U_{X}1+U_{X}2+U_{Y}1+U_{Y}2\right)\cdot \left(L_{P}SD/2\right)$ and $Y=\left(\left(U_{X}2+U_{X}1\right)-\left(U_{Y}1+U_{Y}2\right)\right)/\left(U_{X}1+U_{X}2+U_{Y}1+U_{Y}2\right)\cdot \left(L_{P}SD/2\right)$, where L_PSD is the effective PSD length. This ratio structure provides partial rejection of common-mode intensity fluctuation but remains sensitive to background illumination and channel imbalance.

In order to establish a connection between spot sensing and robot pose measurement, the tracker can be regarded as a measurement station that provides (*L*, *θ*, *φ*) for a tracked target point. The PSD is chiefly concerned with tracking (i.e., maintaining the return spot at the centre), a process which stabilises the angular measurement chain and serves to reduce the probability of losing lock. In instances where multiple target points are measured (for instance, a constellation on the robot end-effector), a rigid-body pose can be estimated through the solution of a point-based registration problem. The present study focuses on the validation of the sensing chain through prototype experiments, while pose-level behaviors are explored through simulations in which PSD, IMU, and encoder data are fused in an EKF framework.

## 3. Prototype System Architecture

The prototype adopts a modular architecture consisting of an optical subsystem, an analogue front end, a data acquisition module, and an FPGA-based processing and control module. Time synchronization among heterogeneous sensors is implemented using a hardware-based strategy on the FPGA platform. The analog signals from the PSD are synchronously sampled by a multi-channel A/D converter driven by a common sampling clock generated by the FPGA. Encoder and IMU data are acquired within the same FPGA clock domain and aligned using a shared hardware trigger signal. All sensor data streams are buffered and assigned deterministic hardware timestamps before being forwarded to the estimation and fusion module. This unified clock and trigger architecture ensures that PSD, encoder, and IMU measurements correspond to the same physical sampling instant, thereby minimizing timing jitter and latency uncertainty. Compared with software-based timestamping, the proposed hardware synchronization approach provides higher temporal consistency and directly supports accurate multi-sensor fusion. The optical subsystem incorporates a dual-frequency He–Ne laser source and a receiver path that generates a spot on the PSD. The analog front end is responsible for the conversion of PSD photocurrents into voltages via I/V conversion, and the subsequent amplification of these voltages to match the A/D input range. The acquisition module samples multiple channels synchronously, and the FPGA performs centroid calculation, filtering, and (in a full closed loop) tracking control output to the motor drivers.

The selection of components is driven by the need to ensure stable interferometric operation and rapid spot feedback. The laser source has a wavelength of approximately 632.991 nm and milliwatt-level output power, thereby enabling interferometry and providing sufficient optical intensity for PSD readout. The PSD device (PSD-2LI10, PSD-2LI10 is manufactured by SiTek Electro Optics AB, located in Partille, Sweden (near Gothenburg)) has an active area of 20 mm × 20 mm and a specified nonlinearity of approximately ±0.8%, with a spectral response spanning 200–1000 nm. A high-speed A/D converter (AD9238BST-65, AD9238BST-65 is manufactured by Analog Devices, Inc. (ADI) in Wilmington, MA, USA) is employed for multi-channel sampling, and the FPGA platform is based on an Altera Cyclone V SoC (Altera Cyclone V SoC is manufactured by Altera (now part of Intel Corporation), located in San Jose, CA, USA), which provides sufficient logic and memory resources for deterministic real-time processing. The actuation is tracked by a direct current (DC) motor (Maxon DC-max26S, Maxon DC-max26S is manufactured by maxon motor ag, located in Sachseln, Switzerland) with a nominal speed of approximately 6260 rotations per minute (rpm) and a rated torque of around 28.5 milli-Newton meters. This motor is suitable for a mirror drive in a laboratory prototype. Key prototype hardware modules and representative parameters are shown in Table 1.

## 4. Methods

This section delineates the data processing and estimation methodologies employed at both the prototype and simulation levels. The prototype’s primary focus is on PSD signal conditioning, centroid computation, and robustness to ambient light. The primary focus of the simulation suite is robot pose estimation, utilising multi-sensor fusion, systematic error modelling, and controller evaluation methodologies. Although the prototype and simulation are not yet coupled into a full closed-loop 6-DoF experimental system, they are designed to be compatible: the same PSD centroid output that is validated in hardware is treated as an optical position observation in the estimator.

### 4.1. PSD Centroid Computation and Calibration

The PSD produces photocurrents at multiple electrodes. After current-to-voltage conversion and amplification, the A/D converter samples the voltages, and the FPGA computes the centroid using a normalized difference-over-sum formula. In implementation, careful attention must be paid to channel gain matching and offset compensation because the centroid is computed from ratios; a small bias in one channel can translate into a systematic position offset.

To evaluate the linear region of the PSD chain, we perform controlled translations around the spot center and fit the measured output using least-squares regression. If the fitted slope differs from 1 or the intercept deviates from zero, a simple affine calibration can be applied: $x_{c}al=a_{x}x_{m}eas+b_{x}$ and $y_{c}al=a_{y}y_{m}eas+b_{y}$. The prototype experiments reported in Section 5 show that within ±0.9 mm spot displacement the system behaves nearly linearly, meaning that such affine calibration is sufficient for the tested range.

### 4.2. Tracking Control Considerations

In order to maintain a high-quality angular readout and to avoid loss of lock, it is essential that a laser tracker keeps the returned beam centred on the receiver. In the prototype, the PSD centroid provides an error signal for mirror actuation. The controller can be implemented as a conventional PID loop because PID is robust to modelling uncertainty and easy to tune. The implementation of more advanced controllers, such as LQR, is contingent upon the availability of a suitable state-space model of the motor–mirror system, in addition to the effective management of constraints, including saturation, friction, and time delay.

In the cited prototype design, an LQR configuration was studied with a scalar control penalty R = 0.1 and a diagonal state penalty Q = diag(1000, 100, 10, 1). This weighting is indicative of a strong preference for minimising primary tracking error, while allowing smaller penalties on higher-order states. The simulations in Section 7 demonstrate that a controller may exhibit suboptimal performance if the assumed model or weight selection is not aligned with the actual dynamics. This underscores the significance of model validation and parameter tuning in ensuring the integrity of the system.

### 4.3. Multi-Sensor Fusion Using EKF

Robot pose estimation is formulated as a discrete-time state estimation problem. The state vector may include position, velocity, and orientation (or incremental angles). The process model predicts state evolution from kinematics and control input, while the measurement model maps the state to sensor observations. Because the measurement equations are typically nonlinear (e.g., due to rotation and coordinate transformations), an extended Kalman filter (EKF) is adopted.

Given state $x_{k}$ and covariance $P_{k}$, the EKF prediction step uses the process model x_{k|k−1} = f(x_{k−1|k−1}, u_{k−1}) and P_{k|k−1} = F_k P_{k−1|k−1} F_k^T + Q, where F_k is the Jacobian of f and Q is the process noise covariance. The update step uses the measurement residual y_k = z_k − h(x_{k|k−1}), innovation covariance $S_{k}=H_{k}P_{k|k-1H_{k}^{T}+R}$, Kalman gain $K_{k}=P_{k|k-1H_{k}^{T}S_{k}^{-1},}$ and updated values x_{k|k=x_{k|k−1+K_ky_k,P_{k|k=├(I−K_kH_k┤)P_{k|k−1}}}}, where H_k is the Jacobian of h and R is the measurement noise covariance.

The simulation suite explicitly varies Q to illustrate convergence behavior. Small Q values indicate high trust in the model and yield smoother estimates, while larger Q values increase responsiveness to measurements but can amplify high-frequency noise. Proper selection of Q and R is therefore scenario dependent. In addition, the simulation includes sensor delays and demonstrates that time synchronization (or delay compensation) is a prerequisite; misaligned timestamps can produce systematic bias that cannot be removed by filtering alone.

### 4.4. Error Modeling and Compensation

The accuracy of laser tracker measurements is influenced by multiple systematic error sources. In the course of the present study, simulations were conducted with the following considerations: The environmental effects that must be considered include the effects of temperature and humidity on the refractive index and thus on the ranging. In addition, there are coordinate transformation errors caused by angular measurement bias or base misalignment. Another effect is target sphere manufacturing deviation, which introduces a constant bias in the effective target centre. Finally, there is base vibration, which introduces time-varying disturbances in both position and angle. In the adopted error modeling framework, foundation vibration is treated as a time-varying disturbance with assumed characteristic frequencies, while other errors such as target sphere deviation are modeled as time-invariant systematic biases within a given trial.

The design of compensation approaches is informed by the identification of error sources. The regression-based correction model that is applied to map environmental variables to distance bias is the most appropriate solution for the issue of temperature and humidity. In the context of coordinate transformation, the utilisation of an angular-error-to-position-error sensitivity model is imperative for the purpose of correcting the mapping. With regard to target sphere deviation, the mean-based calibration is applied relative to a reference artifact. In order to analyse the base vibration, the frequency content is first examined. Following this, targeted filtering (e.g., notch filtering around dominant frequencies) or observer-based suppression is proposed.

### 4.5. Dynamic Environment Adaptability

It is important to note that industrial measurement environments are inherently dynamic. Obstacles, such as moving machinery, can impede the movement of robots, and changes in lighting conditions or obstructions can hinder the clarity of line-of-sight. It is evident that the simulation suite incorporates dynamic obstacle avoidance and illumination compensation. The obstacle avoidance problem is formulated as a path adjustment problem that respects obstacle regions while ensuring that the modified path remains smooth and close to the original. The concept of illumination compensation is modelled as a mapping from light intensity to PSD measurement bias. This approach is informed by the prototype observation that background light has the capacity to compromise the repeatability of measurements, and that the combination of optical filtering and compensation can enhance the robustness of the measurement process.

## 5. Prototype Bench Experiments

Prototype experiments are conducted to validate the feasibility of the PSD sensing chain and to quantify its performance under controlled conditions. The following three experiments are reported: The primary focus of this study is the investigation of the linearity of the PSD readout following signal conditioning and A/D sampling under controlled X/Y translations. A secondary focus is the examination of the sensitivity of the measured centroid to alterations in the laser head–PSD distance along the Z axis. Finally, the study will address the influence of background light and the potential benefits of incorporating an interference filter.

The experimental setup consists of a laser source mounted on a precision three-dimensional translation stage, a fixed PSD receiver module, analog conditioning circuits, and an FPGA-based acquisition and processing board. The PSD plane is adjusted to be approximately perpendicular to the incoming beam. In the context of translation experiments, the initial placement of the spot is centred, with subsequent translations of the stage executed in incremental steps. Concurrently, the FPGA (field-programmable gate array) performs real-time computations of centroid coordinates.

### 5.1. Linearity Under Controlled Spot Translation

The laser head is translated along X and Y around the PSD center with a step size of 0.1 mm, covering a range of ±1 mm. The analysis focuses on the interval ± 0.9 mm where stable spot formation and reliable readout are maintained. Linear regression is applied separately for positive and negative directions to assess hysteresis and asymmetry. Table 2 summarizes the fitted results.

The fitted R^2^ values around 0.998–0.999 indicate near-ideal linear behavior in the tested range. The slopes are close to unity, while small differences between directions suggest minor asymmetry that can originate from channel gain mismatch, small non-orthogonality, or translation-stage hysteresis. For tracking control, the observed linearity is sufficient because the control objective is to maintain the spot near the center; in that regime, an affine calibration can remove residual bias.

### 5.2. Influence of Laser Head–PSD Distance

To evaluate geometric sensitivity, the laser head is translated along the Z axis relative to the PSD. The Z position is varied from 0 to 10 mm with step size 1 mm. Linear regression is applied to the measured X and Y centroid outputs as a function of Z. Table 3 reports the fitted relationships.

The results show that X is relatively insensitive to Z within the tested range, while Y shows a larger slope and weaker linearity. This asymmetry likely reflects residual misalignment in the optical setup (e.g., slight pitch/yaw offset). The experiment highlights a practical caution: spot-plane linearity alone does not guarantee geometric invariance. For high-accuracy deployment, mechanical alignment should be improved, and if necessary, distance-dependent calibration terms can be introduced to correct Z-coupled bias.

### 5.3. Background Light Influence and Interference Filter Validation

Background illumination introduces additional photocurrent in the PSD and can change the effective centroid output. Two mitigation families are common: electrical modulation/demodulation (effective but more complex) and optical filtering (simple but partial). In this prototype, an interference optical filter is installed in front of the PSD to suppress ambient light outside the laser wavelength band. Repeatability is evaluated by scanning the spot along one axis and computing the standard deviation of repeated measurements.

With the optical filter, the reported displacement statistic yields a standard deviation σ = 0.0032 in the test, whereas without the filter σ increases to 0.0239. Although the mean displacement differs slightly, the reduction in dispersion demonstrates that optical filtering strongly improves measurement stability under ambient conditions. Table 4 summarizes the comparison.

In addition to improving repeatability, optical filtering also improves robustness for closed-loop tracking because the controller sees a cleaner error signal. In future work, combining optical filtering with modulation-based demodulation may further suppress background influence and allow robust operation under stronger illumination changes.

## 6. Simulation Suite for Pose Estimation and Robustness

In order to complement prototype validation and to explore system-level behaviour, a MATLAB2024a/Simulink simulation suite was implemented. This integrates three sensor modalities (PSD, IMU, and encoder) and a set of error models and control strategies. The simulations have been designed to be interpretable and to isolate the effect of specific factors, including: process noise covariance selection, sensor fusion benefit for velocity and position, noise intensity stress tests, time synchronisation, computational scalability, environmental compensation, coordinate transformation sensitivity, vibration characterisation, controller comparison, and dynamic environment adaptation.

The ensuing subsections present the simulation results utilising the provided figures. It should be noted that, unless explicitly stated otherwise, the figures presented herein illustrate representative trajectories with additive Gaussian white noise. The parameters of the estimators and controllers have been selected to reproduce the observed trends. The objective is to furnish engineering insights (i.e., the factors that facilitate success and those that are detrimental to it, and the underlying reasons for each) as opposed to asserting a set of parameters that is universally optimal.

### 6.1. Multi-Sensor Fusion Performance

**Figure 1:**
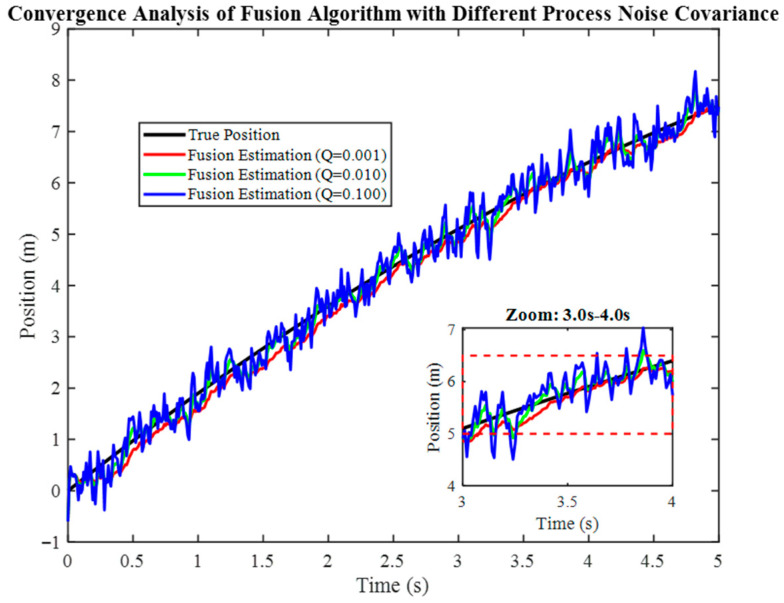
Convergence analysis of the fusion algorithm under different process noise covariance settings.

Figure 1 examines convergence of the fused position estimate under three process noise covariance settings (Q = 0.001, 0.010, 0.100). The true position displays a consistent upward trend. When Q is set to 0.001, the fused estimate displays negligible variation and closely aligns with the truth, suggesting a high degree of confidence in the model. When Q is set to 0.010, the estimate demonstrates stability, exhibiting only moderate fluctuations. When Q is set at 0.100, fluctuations become more pronounced and the estimate becomes more noisy, particularly at later times. However, it remains within the bounds and does not diverge. This phenomenon exemplifies the fundamental trade-off inherent in any estimation process: the choice between achieving a smoother estimate through a smaller Q and the risk of under-reacting to abrupt motion, versus the adoption of a larger Q to achieve a faster response but with the concomitant admission of more measurement noise.

**Figure 2:**
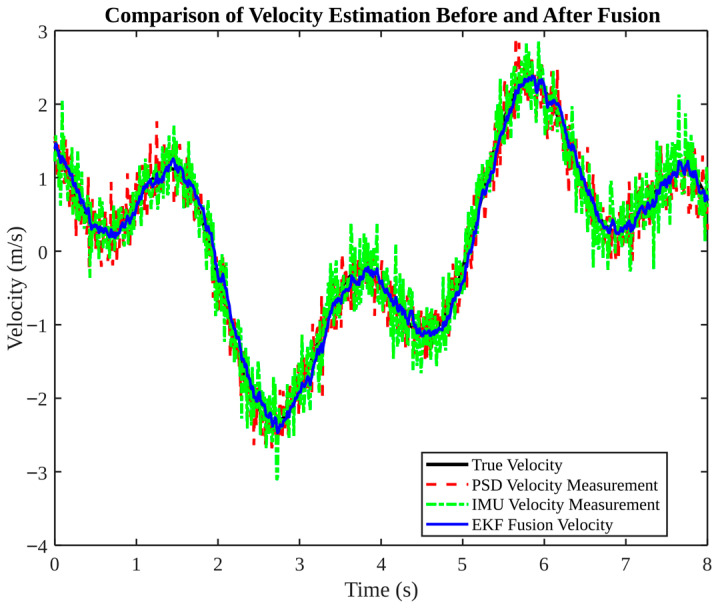
Velocity estimation comparison before and after fusion (PSD vs. IMU vs. EKF).

Figure 2 compares velocity estimation before and after fusion. The velocities derived from raw PSD and IMU data exhibit significant fluctuations. IMU data demonstrates a tendency to produce frequent spikes, a phenomenon that is commonly observed during the integration or differentiation of acceleration noise. EKF-fused velocity is shown to be significantly smoother and to follow the true velocity trend closely, thus demonstrating that complementary sensor information can suppress random noise and improve dynamic estimation.

**Figure 3:**
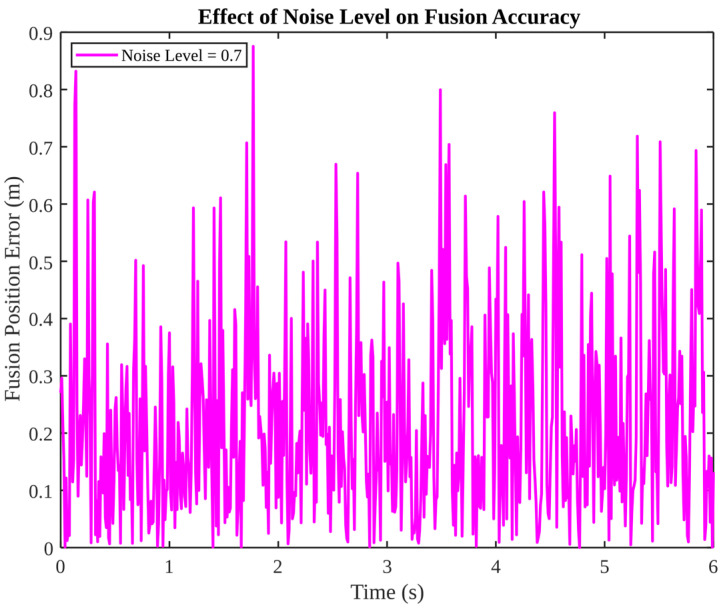
Effect of noise level on fusion accuracy (noise level = 0.7).

As illustrated in Figure 3, a high-noise-intensity case is presented, with a noise level of 0.7. The fusion position error oscillates at a high frequency but remains within a bounded interval, indicating that the estimator remains stable even under conditions of severe noise. The figure suggests that further enhancement of this regime would necessitate either an improvement in the signal-to-noise ratio of sensors or the adaptive tuning of covariance matrices to mitigate the impact of substantial noise bursts.

**Figure 4:**
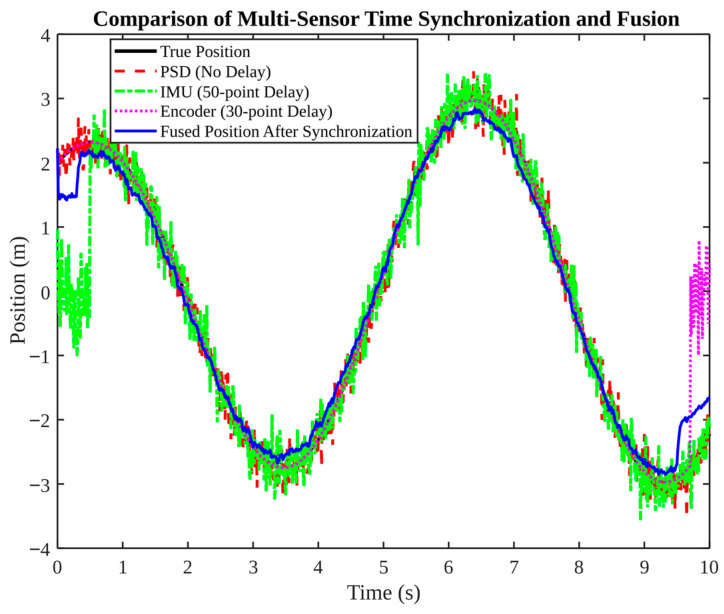
Comparison of multi-sensor time synchronization and fusion results.

As demonstrated in Figure 4, time synchronisation is a pivotal aspect of the system. The three sensors exhibit varying delays (PSD: no delay; IMU: 50-sample delay; encoder: 30-sample delay). In the absence of synchronization, delayed measurements have the potential to introduce bias and discontinuities. Following synchronisation, the fused position closely follows the true position, with delay-induced deviations largely disappearing. The result demonstrates that time alignment is not a matter of choice: a filter can only fuse what it receives. If the signals refer to different physical times, the fused estimate is inherently biased.

**Figure 5:**
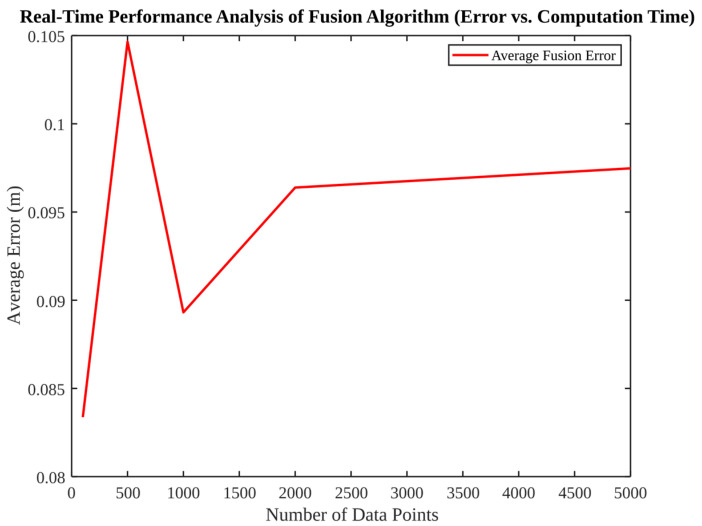
Real-time performance analysis of fusion (average error vs. number of data points).

Figure 5 evaluates scalability by plotting average fusion error versus number of data points. The mean error fluctuates at low data volumes but stabilises at approximately 0.095 m after approximately 2000 samples, suggesting that the algorithm reaches a steady-state accuracy determined by model consistency and sensor noise floor. It is important to note that the error does not increase with the length of the data, which indicates that the estimator maintains numerical stability for extended sequences.

### 6.2. Systematic Error Analysis and Compensation

**Figure 6:**
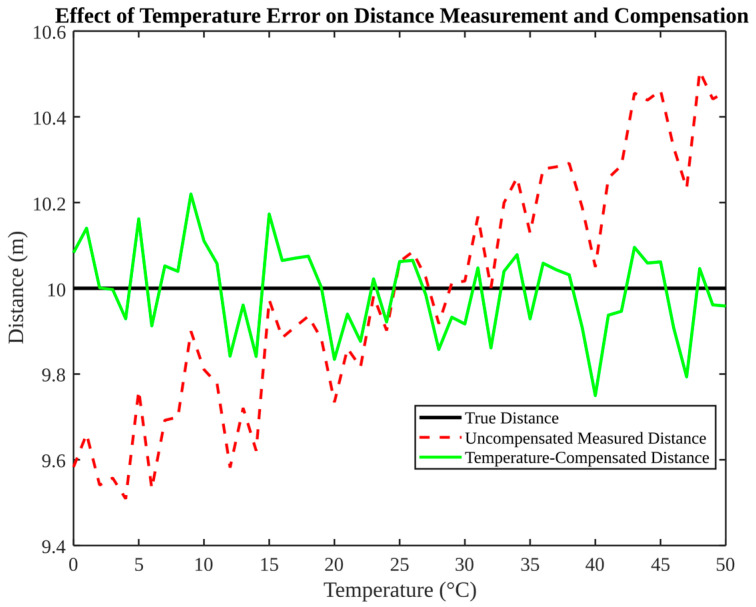
Effect of temperature error on distance measurement and compensation.

As illustrated in Figure 6, this phenomenon is characterised by the occurrence of distance drift and subsequent compensation in response to variations in temperature. The true distance remains approximately 10 m, while the uncompensated measurement exhibits a drift with temperature, reaching a deviation of more than 0.5 m at high temperatures. Subsequent to the implementation of compensation, the distance that has undergone correction exhibits a high degree of overlap with the true distance, with only minor random fluctuations observed. This result demonstrates the capacity of environmental compensation to transform a substantial systematic bias into a residual noise process with a mean close to zero.

**Figure 7:**
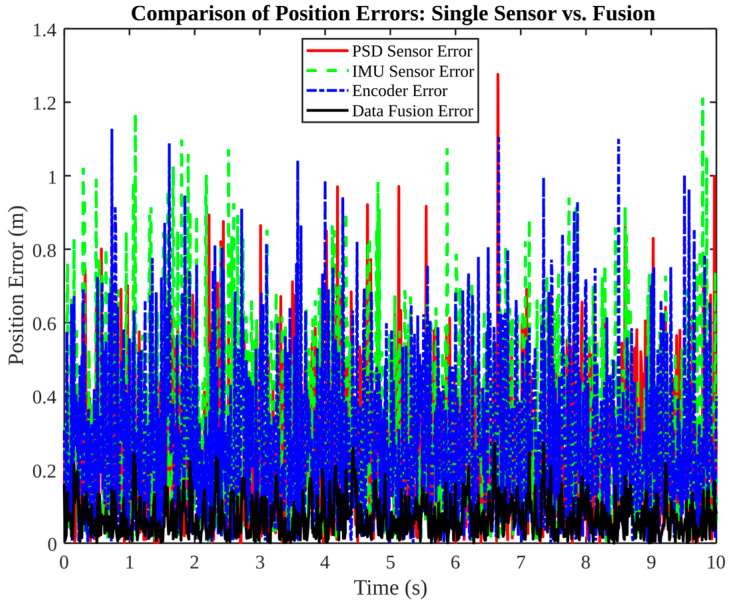
Comparison of position errors: single sensor vs. fusion.

Figure 7 compares single-sensor position errors (PSD, IMU, encoder) against the fused error. Errors in the IMU and encoder have been observed to exceed 1.0 m, while those in the PSD are smaller but still fluctuate. The fusion process has been shown to reduce the error envelope to within approximately 0.2 m and to produce a smoother curve, thus confirming the “complementarity” benefit in position estimation.

**Figure 8:**
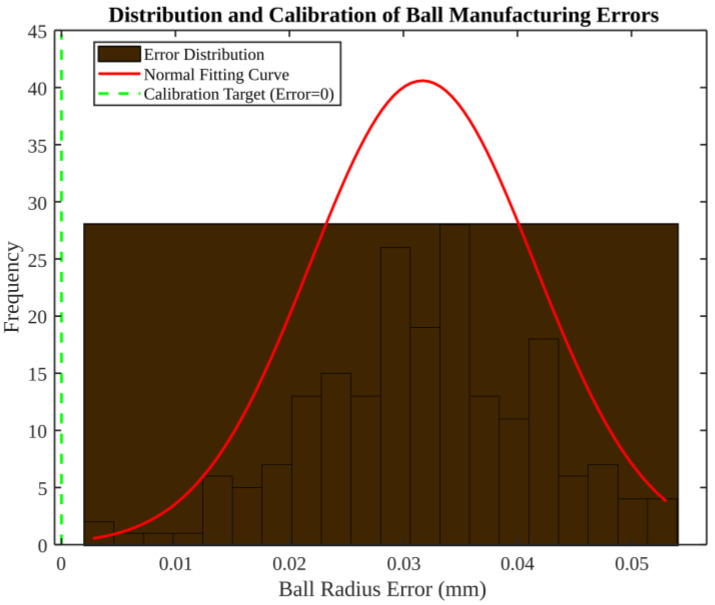
Distribution and calibration of ball manufacturing errors (target sphere).

As illustrated in Figure 8, the model exhibits manufacturing errors in the target sphere, specifically, a ball radius error. The histogram indicates an approximately normal distribution, with most errors concentrated around 0.02–0.04 mm and a mean near 0.03 mm. The calibration target, which is defined as the theoretical value at which the measurement is to be aligned, is illustrated as being at zero error. It is evident that a calibration based on the mean can shift the distribution towards zero. It is imperative to acknowledge the significance of even the minutest artefact errors in precision metrology, as they can have a substantial impact on the accuracy of the results. Consequently, explicit calibration of the target geometry is a pragmatic necessity. Figure 8. Distribution of target sphere (SMR) manufacturing deviations and its calibration implication. In simulations, one deviation value is sampled once per trial from this distribution and held constant throughout the time-domain run.

**Figure 9:**
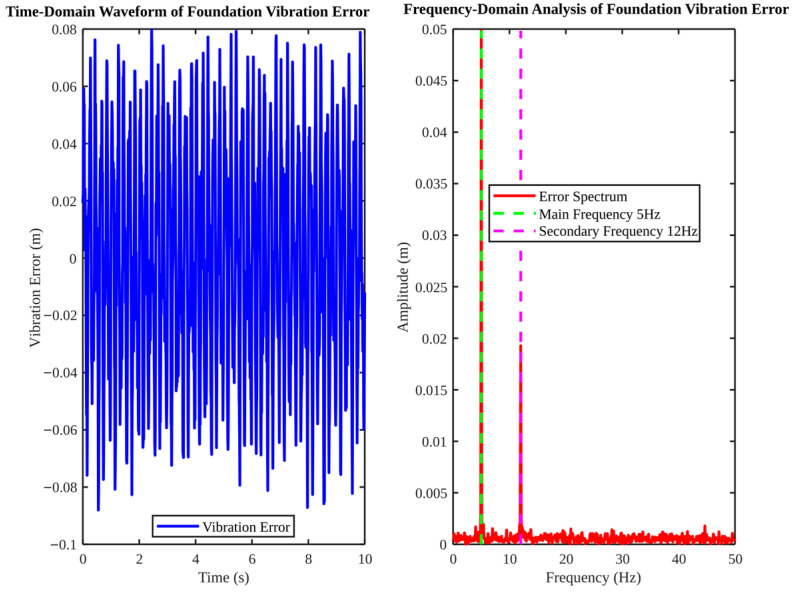
Foundation vibration error in time and frequency domains.

Figure 9 provides an analysis of base vibration in both time and frequency domains. The time-domain vibration error fluctuates within approximately ±0.1 m. The fast Fourier transform (FFT) identifies dominant frequency components around 5 Hz (main) and 12 Hz (secondary). This provides a concrete basis for targeted vibration suppression, such as notch filtering or model-based disturbance observers. Figure 9. Simulated foundation vibration error in the time and frequency domains. The dominant frequency components (5 Hz and 12 Hz) are assumed to be representative of typical low-frequency industrial foundation vibrations.

**Figure 10:**
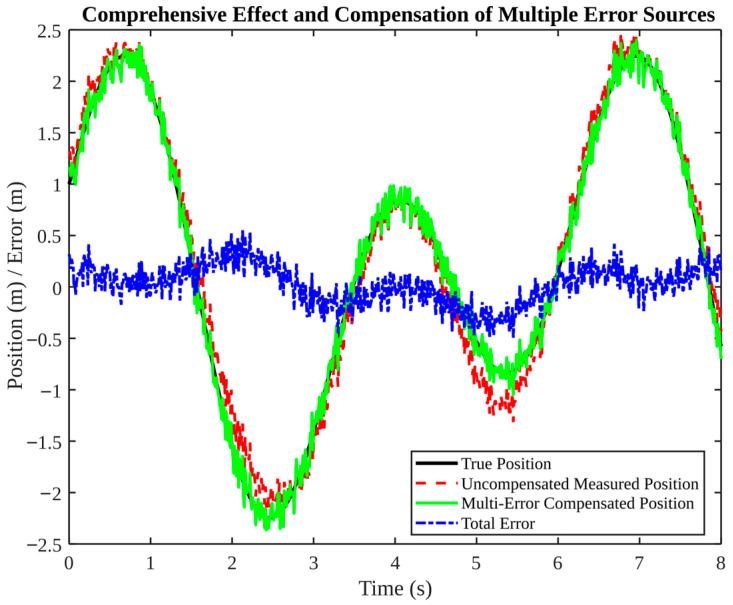
Comprehensive effect and compensation of multiple error sources.

In this time-domain simulation, the target sphere deviation is applied as a fixed systematic bias for the entire trial (consistent with a physical SMR), rather than as a time-varying random noise term. As demonstrated in Figure 10, this effect is compounded by multiple error sources, and joint compensation is shown to be beneficial. It is evident that the uncompensated measured position deviates significantly from the true trajectory, with errors exceeding 2 m. However, upon the application of multi-error compensation, the corrected position exhibits a close alignment with the true trajectory. This outcome is consistent with real-world systems: correcting a single error source may yield limited improvement if other biases predominate; layered compensation is often required. Modeling assumption for target sphere deviation: In physical reality, a given SMR/target sphere has a fixed geometric deviation (e.g., radius/center offset) that remains constant over time. Therefore, in our simulations, the target sphere deviation is modeled as a constant systematic bias within each trial. Specifically, a bias value is sampled once from the manufacturing-error distribution (Figure 8) at the beginning of a trial and then kept constant throughout the entire time-domain simulation (e.g., Figure 10). Across multiple trials, different fixed bias values are used to represent sphere-to-sphere manufacturing variability.

**Figure 11:**
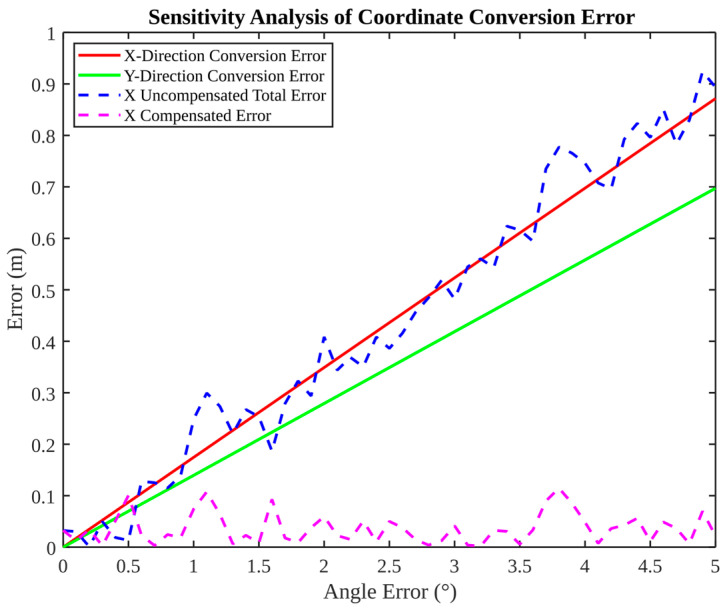
Sensitivity analysis of coordinate conversion error and compensation.

Figure 11 examines the relationship between coordinate conversion sensitivity and angular error. It is evident from the data that both X- and Y-direction conversion errors increase approximately linearly with angle error, thus illustrating high sensitivity. Following the implementation of compensation, the X-direction compensated error remained at a negligible level, exhibiting only minor fluctuations. This finding suggests that an error mapping model can effectively mitigate transformation-induced bias.

**Figure 12:**
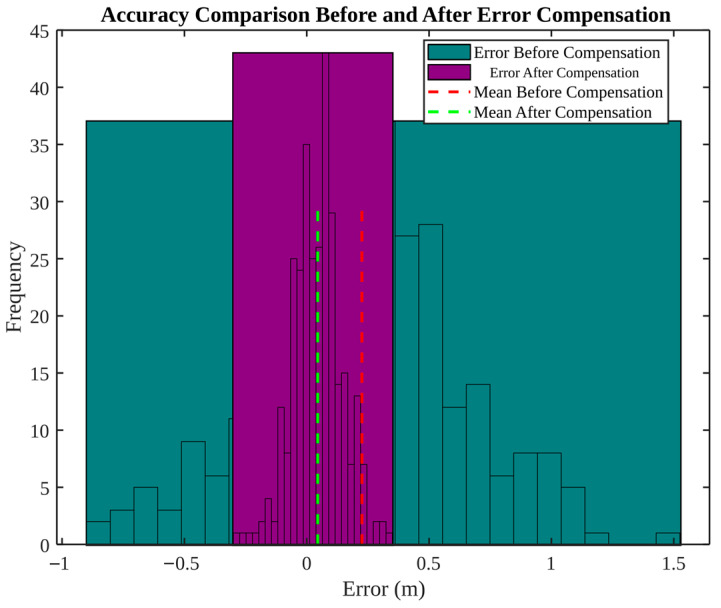
Accuracy comparison before and after error compensation (error distribution).

Figure 12 provides a statistical summary of compensation benefit via histograms. Prior to the implementation of compensation, errors are distributed in a relatively broad manner (approximately −1 to 1.5 m) with a nonzero mean. Following the implementation of compensation, the distribution becomes concentrated near zero, with a narrower spread (approximately −0.5 to 0.5 m) and mean close to zero. The distribution-level evaluation is advantageous in that it reveals outliers and heavy tails that may be obscured by a single RMSE number.

### 6.3. Control Algorithm Comparison

**Figure 13:**
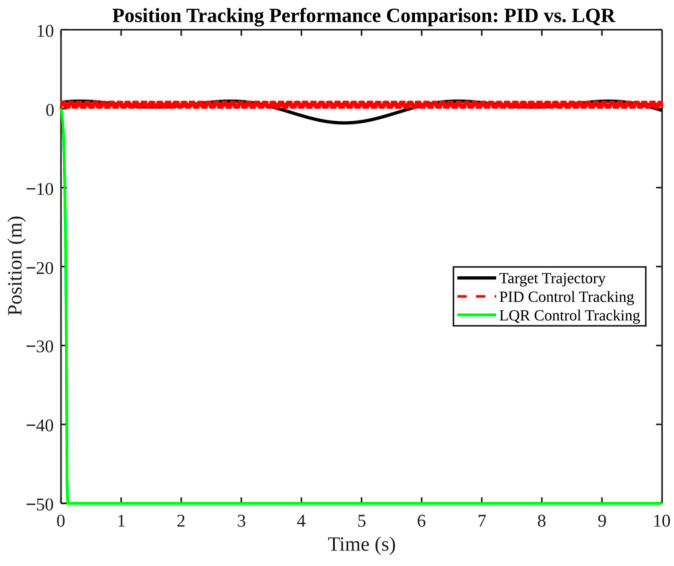
Position tracking performance comparison: PID vs. LQR.

**Figure 14:**
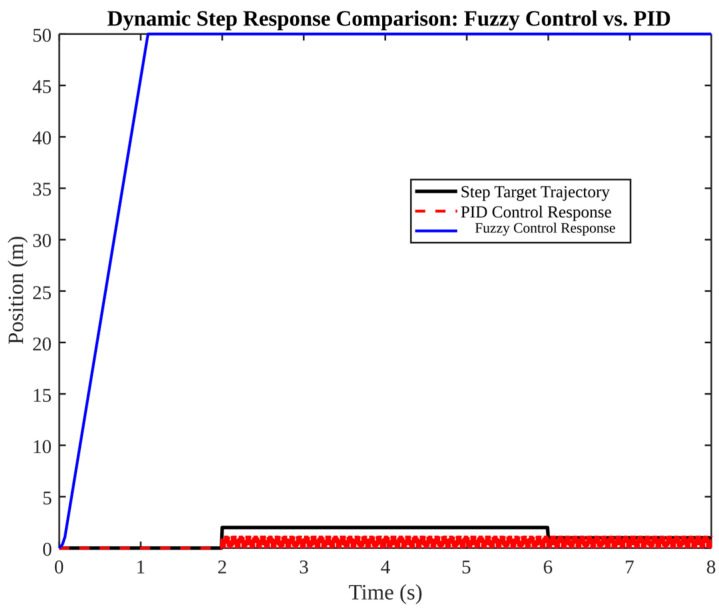
Dynamic step response comparison: fuzzy control vs. PID.

**Figure 15:**
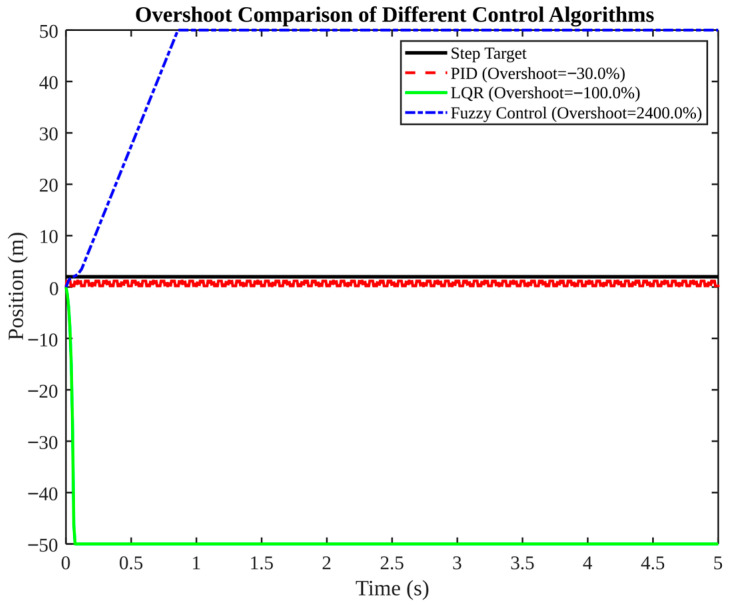
Overshoot comparison of different control algorithms.

**Figure 16:**
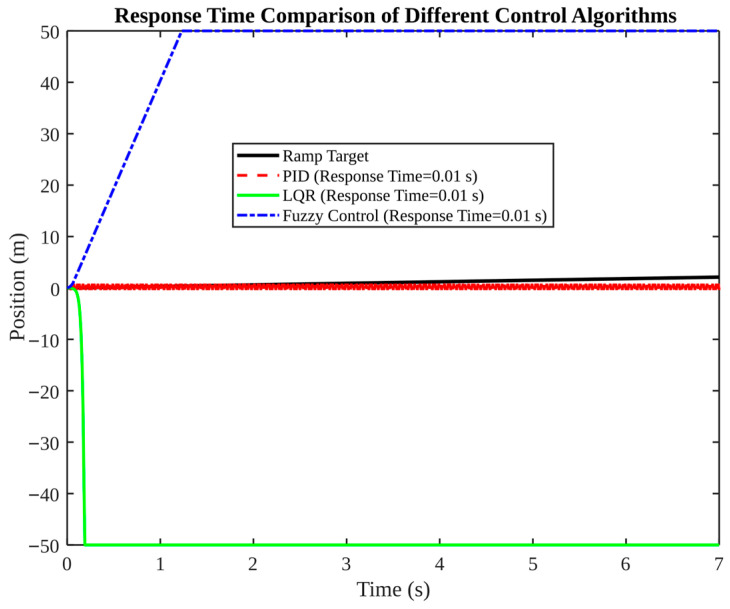
Response time comparison of different control algorithms.

**Figure 17:**
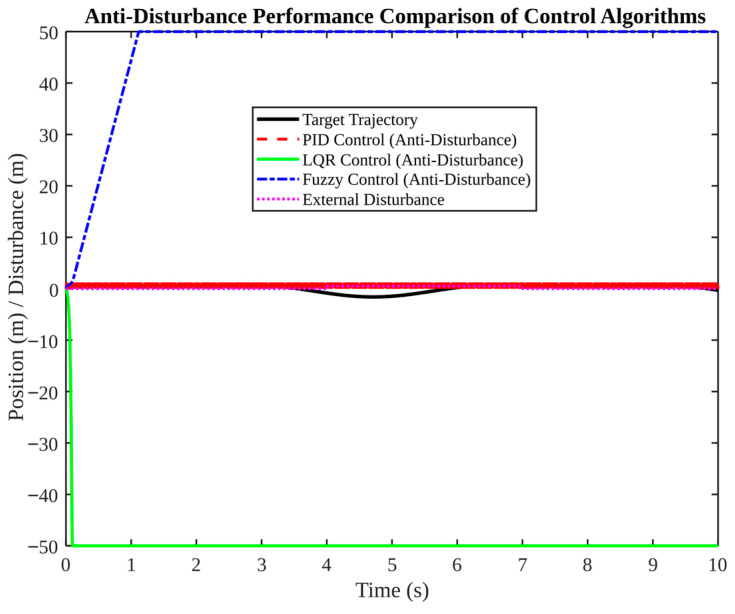
Anti-disturbance performance comparison under external disturbance.

Figure 13, Figure 14, Figure 15, Figure 16 and Figure 17 present a comparative analysis of PID, LQR, and fuzzy control in pose tracking scenarios. The objective of these experiments is not to establish a universally superior controller; rather, they serve to illustrate how controller performance is contingent on model fidelity and tuning.

Figure 13 compares PID and LQR tracking. PID demonstrates a high degree of convergence on the target trajectory, with minor oscillations. In contrast, LQR exhibits a significant divergence, reaching a minimum of −50 m. These findings suggest that the LQR controller, as configured, is not compatible with the simulated plant or constraints. In practice, LQR necessitates an accurate model and appropriate weighting; otherwise, the resulting feedback can be unstable or saturate actuators.

Figure 14 compares fuzzy control and PID under a step-like target. The fuzzy control system demonstrates a rapid rise time, reaching the target within approximately 1 s. In contrast, the PID controller remains near zero in this configuration, indicating inadequate tuning for step tracking. This demonstrates that controller suitability is trajectory dependent: a controller tuned for smooth tracking may underperform under abrupt changes such as reacquisition after occlusion.

Figure 15 compares overshoot among controllers. PID exhibits a modest negative overshoot of −30%, LQR exhibits −100% overshoot (diverging), and fuzzy control exhibits extreme overshoot of 2400%. The figure indicates specific tuning directions: the incorporation of overshoot suppression rules for fuzzy control and the recalibration of weight matrices for LQR to circumvent instability and substantial bias.

As demonstrated in Figure 16, response time alone is not a sufficient metric. Despite the fact that all controllers report the same nominal response time (0.01 s), it is evident that only PID is capable of accurately tracking the target in the plotted case. It is therefore essential that controller evaluation includes both transient metrics (e.g., rise time, overshoot) and steady-state accuracy and stability under noise and constraints.

The anti-disturbance performance of the system is evaluated in Figure 17. In the presence of an external disturbance, the PID controller maintains proximity to the target, while the LQR and fuzzy control systems deviate significantly. In the context of early-stage prototypes, where the models are less certain, PID can serve as a reliable baseline. However, it has been demonstrated that more advanced controllers can only outperform PID in instances where plant modelling, constraint handling, and tuning are adequately addressed.

### 6.4. Dynamic Environment Adaptation

**Figure 18:**
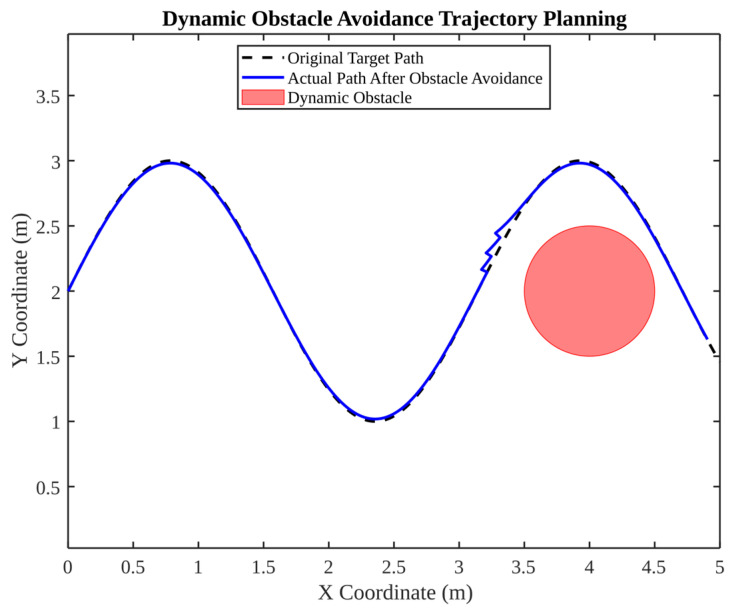
Dynamic obstacle avoidance trajectory planning.

**Figure 19:**
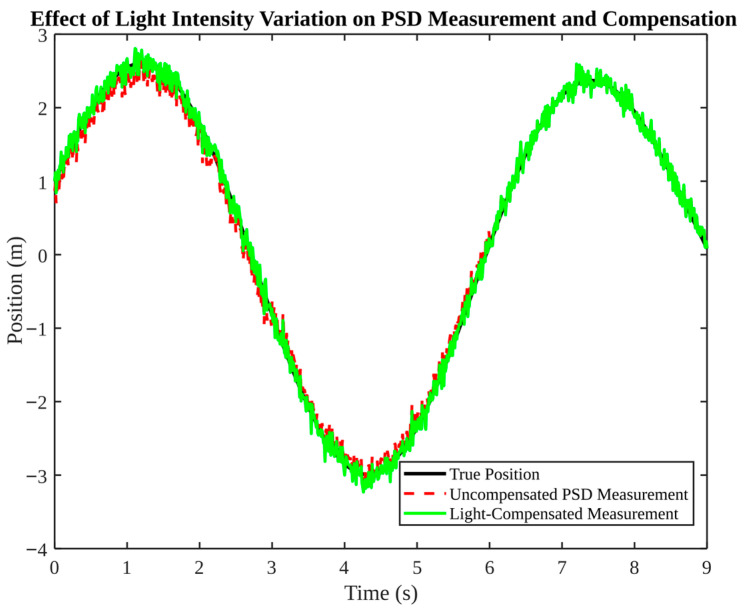
Effect of light intensity variation on PSD measurement and compensation.

**Figure 20:**
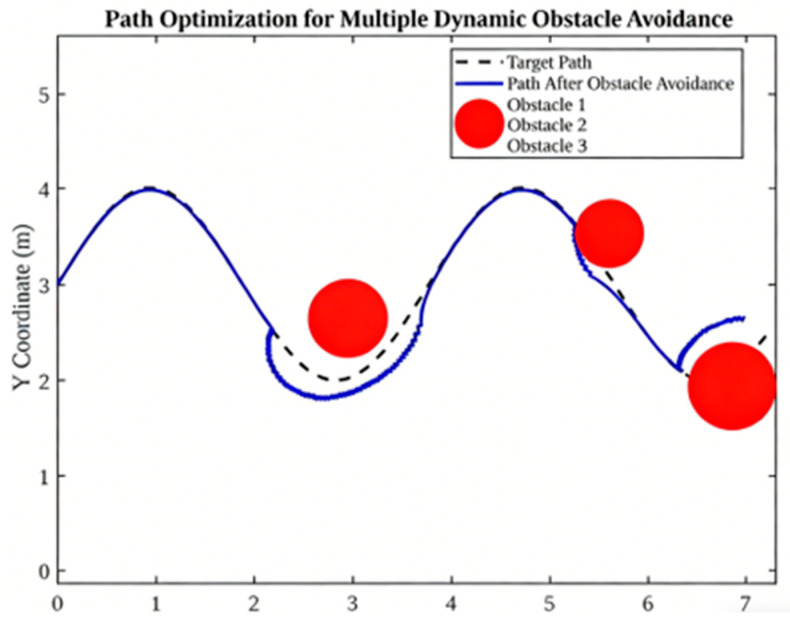
Path optimization for multiple dynamic obstacle avoidance.

**Figure 21:**
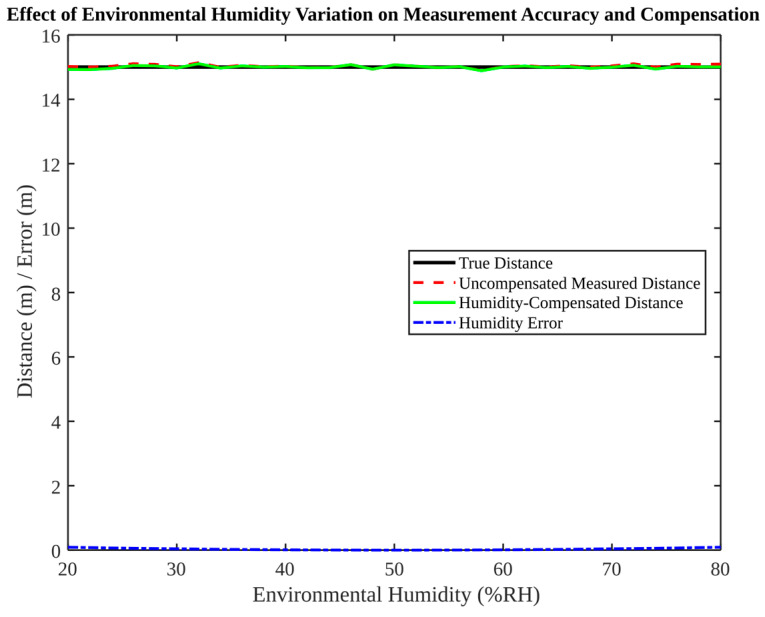
Effect of environmental humidity variation on measurement accuracy and compensation.

The subsequent Figure 18, Figure 19, Figure 20 and Figure 21 explore dynamic environment factors, including moving obstacles, illumination changes and humidity variation. Despite the simplification of these simulations, they provide a valuable framework for the design of resilient field systems.

As illustrated in Figure 18, this dynamic obstacle avoidance scenario is a critical component of the overall system. The original path intersects an obstacle region, while the adjusted path circumvents the obstacle in a smooth and consistent manner, maintaining proximity to the original trend. The relevance of such path planning is evidenced by the necessity of maintaining line-of-sight and avoiding collisions for the purpose of ensuring the safety of robot metrology.

As illustrated in Figure 19, the impact of light intensity variations on PSD measurement and compensation is evident. In the absence of such compensation, discrepancies arise between the measured and actual positions, a phenomenon particularly pronounced during illumination transitions. Subsequent to the implementation of compensation, the rectified measurement exhibits a close congruence with the truth. The result corroborates the initial observation that ambient light can compromise repeatability; the integration of hardware filtration with software compensation provides a resilient approach.

As illustrated in Figure 20, the system has been developed to encompass the avoidance of multiple dynamic obstacles. The optimised trajectory incorporates local detours and micro-adjustments to circumvent three obstacles while preserving overall smoothness. This finding underscores the importance of adaptable planning methodologies in workspaces characterised by disorder.

Figure 21 provides an analysis of humidity variation and compensation. Within the range of 20% to 80% relative humidity, the humidity-induced distance error remains negligible in the model. However, the implementation of compensation further mitigates residual deviation. Although humidity may be a weaker factor than temperature in some scenarios, it can still contribute to refractive index variation and should be included in comprehensive environmental compensation when high accuracy is required. The statistical summary of positioning errors under different conditions is shown in Table 5.

## 7. Discussion

The amalgamation of prototype and simulation results offers several practical insights. Firstly, it is imperative to acknowledge the pivotal role of the spot sensing chain in this context. The near-linear power spectral density (PSD) readout within ±0.9 mm, and the strong repeatability improvement from an interference filter, suggest that the sensing chain can provide a stable tracking error signal. However, the Z-sensitivity experiment demonstrates that geometric misalignment can induce distance-coupled bias. This suggests that mechanical alignment and distance-aware calibration should be considered during the early stages of system integration.

Secondly, simulations demonstrate that the performance of estimators is contingent not only on system engineering, but also on mathematical principles. The EKF fusion performs optimally when sensor signals are time-aligned; in the absence of synchronisation, delays introduce systematic bias that filtering cannot “average out.” In the context of real systems, it is imperative to measure and either minimise or explicitly model timestamping, buffering, and communication latency. A robust implementation often necessitates a time synchronisation strategy (e.g., hardware triggers, clock discipline, or software delay compensation) in conjunction with the filter itself.

Thirdly, systematic error compensation is optimally approached as a layered problem. The factors of temperature, coordinate transformation, target sphere deviation and vibration can each assume a dominant role, depending on the environmental factors present. The multi-error compensation simulation demonstrates that joint compensation can reduce meter-level deviations to near-zero-mean residuals. The translation of this into a field system would necessitate the incorporation of environmental sensors for temperature, pressure, and humidity. Furthermore, the implementation of calibration procedures for base alignment and angular encoders would be essential. In addition, the characterisation of target artifacts would be crucial, as would the monitoring and mitigation of vibrations. It is imperative that compensation models be validated under realistic conditions; otherwise, they risk introducing new forms of bias.

Finally, it is imperative that controller selection reflects modelling uncertainty and operational constraints. PID performs robustly in the simulations, whereas LQR and fuzzy control demonstrate failure modes when tuning is not matched to the plant and trajectory. In practice, a rational approach is to adopt a well-tuned PID controller as a baseline and then incrementally incorporate model-based control only after system identification and constraint handling (saturation, friction, rate limits) have been addressed. In the context of fuzzy control, it is imperative that rule-based design explicitly incorporates overshoot suppression and robustness to disturbances.

The present study does not focus on a specific commercial robot model. Instead, the simulation framework employs a generic rigid-body robot representation with an encoder, an IMU, and external optical measurements. As a result, the proposed sensing, fusion, and compensation methodology is not tied to a particular robot architecture and can be generalized to different industrial robots, including serial manipulators and large-scale gantry systems. While absolute numerical performance may vary with robot stiffness, kinematic configuration, and sensor quality, the observed trends in sensor fusion, time synchronization, and layered error compensation are broadly applicable.

The present study is subject to several limitations. Firstly, a full closed-loop experimental demonstration of 3D point reconstruction and 6-DoF robot pose estimation using the prototype hardware has not been conducted. The simulations provide valuable system-level insights; however, future work should integrate the interferometric ranging link, angle measurement, and PSD-based tracking into a complete experimental platform, with quantitative pose accuracy evaluation against a calibrated reference.

## 8. Conclusions

This paper presented a laser-tracker-oriented robot pose measurement framework that combines interferometric ranging with PSD-based beam-spot sensing and FPGA processing. Prototype bench experiments validated the core PSD sensing chain, demonstrating near-linear behavior within ±0.9 mm spot displacement and significant repeatability improvement when an interference optical filter is used to suppress background illumination. A comprehensive simulation suite then explored system-level pose estimation and robustness, showing that EKF-based fusion of PSD, IMU, and encoder data can suppress noise and drift, that time synchronization is a prerequisite for accurate fusion, and that layered error compensation can dramatically reduce systematic deviations. Control simulations highlighted the robustness of PID under uncertainty and the tuning sensitivity of LQR and fuzzy control. Together, these results provide practical guidance and a development roadmap toward robust tracker-based robot pose metrology in industrial environments.

## Figures and Tables

**Table 1:** Key prototype hardware modules and representative parameters.

Module	Model	Representative Parameters
Laser source	Dual-frequency He–Ne	λ ≈ 632.991 nm; output power 1.2–1.5 mW; spot diameter ≈ 0.7 mm; RMS noise < 0.1% (30 Hz–10 MHz); frequency stability ± 1.0 MHz/min, ±2.0 MHz/h
Spot sensor	PSD-2LI10 (pillow-type 2D PSD)	Active area 20 mm × 20 mm; nonlinearity ±0.8%; reverse bias 15 V; spectral response 200–1000 nm; dark current ≈ 1.0 nA; capacitance ≈ 300 pF
A/D converter	AD9238BST-65	Multi-channel sampling; integrated sampling clock and reset control (used for synchronous acquisition in the prototype)
FPGA platform	Altera Cyclone V SoC (5CSEMA5F31C6N)	≈85 k logic elements; ≈4450 KB embedded memory; PLL and global clock resources for deterministic sampling/control
Tracking motor	maxon DC-max26S	Nominal speed ≈ 6260 rpm; rated torque ≈ 28.5 milli-Newton meters; nominal current ≈ 0.46 A
Analog front end	I/V and amplification stages	Operational amplifier-based current-to-voltage conversion and gain matching; designed to support centroid computation via normalized difference-over-sum

**Table 2:** Linearity fitting results for PSD centroid readout within ±0.9 mm.

Axis/Direction	Fit (y = ax + b)	R^2^	Range (mm)
X (positive)	a = 1.015, b = 0.007	0.999	−0.9 to +0.9
X (negative)	a = 1.001, b = 0.001	0.998	−0.9 to +0.9
Y (positive)	a = 0.989, b = 0.002	0.998	−0.9 to +0.9
Y (negative)	a = 0.983, b = −0.006	0.998	−0.9 to +0.9

**Table 3:** Sensitivity of measured centroid coordinates to the laser head–PSD distance (Z).

Output	Fit	R^2^
x (z)	x = −0.0006 z − 0.124	0.9298
y (z)	y = −0.0056 z − 0.923	0.7862

**Table 4:** Repeatability comparison with and without an interference optical filter.

Condition	Mean Metric L	Std. Dev. σ
With interference filter	0.0856	0.0032
Without interference filter	0.0768	0.0239

**Table 5:** Statistical summary of positioning errors under different conditions.

Measurement Condition	Mean Error (m)	RMS Error (m)	Maximum Error (m)
Single PSD measurement	0.42	0.51	0.93
Single IMU estimation	0.88	1.02	1.76
Single encoder estimation	0.67	0.84	1.45
Multi-sensor fusion (EKF)	0.09	0.12	0.21
Fusion with error compensation	0.04	0.06	0.11

## Data Availability

Prototype bench results and simulation figures are included in this manuscript. Additional implementation details can be provided upon reasonable request.
